# Carcinome neuroendocrine du col utérin: à propos d’un cas avec revue de la littérature

**DOI:** 10.11604/pamj.2017.27.82.10902

**Published:** 2017-06-02

**Authors:** Soufiane Baggar, Hajar Ouahbi, Meryem Azegrar, Fatima Zahra El M’rabet, Samia Arifi, Nawfel Mellas

**Affiliations:** 1 Service d’Oncologie Médicale CHU Hassan II, Fès, Maroc

**Keywords:** Carcinome à petites cellules neuroendocrine, col utérin, radiothérapie chimiothérapie, Small cell neuroendocrine carcinoma, cervix, radiation therapy, chemotherapy

## Abstract

Les carcinomes neuroendocrines à petites cellules gynécologiques sont inhabituels, et ils ne représentent que 2% des tumeurs du col utérin. Compte tenu de leur rareté et de l’absence d’essais randomisés, la prise en charge diagnostique et thérapeutique de ces tumeurs est difficile et est essentiellement calquée sur celle des tumeurs neuroendocrines pulmonaires. A l’instar de ces dernières, et malgré un traitement multimodal, leur pronostic reste défavorable. Nous rapportons un nouveau cas de carcinome neuroendocrine du col à petites cellules et à travers les données de la littérature nous mettons le point sur les différents aspects de cette entité rare.

## Abstract (en)

Small cell neuroendocrine carcinomas of the gynecologic tract are unusual, accounting for only 2% of the cervical cancers. Given the rarity of these tumors and the absence of randomized trials, their diagnosis and treatment programmes are difficult and are essentially based on those of neuroendocrine tumors of the lung. As in the case of the neuroendocrine tumors of the lung and despite multimodal treatment they are associated with a poor prognosis. We here report a new case of small cell neuroendocrine carcinoma of the cervix and, throught a literature review, we highlight the various aspects of this rare entity.

## Introduction

Le carcinome à petites cellules est un type de cancer neuroendocrinien qui prend naissance dans les cellules du système neuroendocrinien. Il a tendance à être agressif et on l´associe à un pronostic moins encourageant, même si on le diagnostique à un stade précoce. Une observation récente nous a permis de revoir la littérature concernant cette entité rarissime.

## Patient et observation

Madame R.R, âgée de 47ans, 2^ème^ geste 2^ème^ paire, avec antécédent de cancer du colon chez son oncle et son grand père paternel, un cancer du col utérin chez une tante maternelle. Le premier signe clinique alarmant était des métrorragies de faible abondance avec dyspareunie, sans signes digestifs ou urinaires le tout évoluait dans un contexte de conservation de l’état général. L’examen au speculum révèle une volumineuse masse cervicale hémorragique de la lèvre antérieure et postérieure du col, d’environ 5cm de grand axe. Aux touchers pelviens l’utérus est légèrement augmenté de taille et les paramètres sont libres.

Les biopsies réalisées à ce niveau concluaient à une prolifération tumorale maligne de nature carcinomateuse constituée de cellules de taille petite ou moyenne, à cytoplasmes éosinophiles modérément abondant dotées de noyaux fortement anisocaryotiques fortement en mitose. Ces cellules se disposent en massifs ou nappes diffuses volontiers centrés par de la nécrose sans différenciation glandulaire ou malpighienne notable. Ces massifs carcinomateux se disposent au sein d’un stroma fibrocollagène modérément abondant.

**Figure 1:**
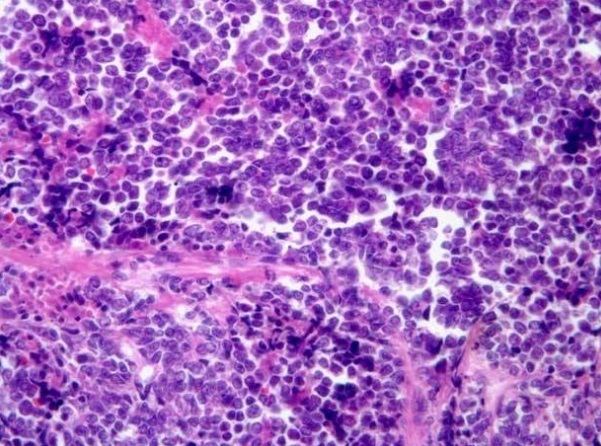
Prolifération tumorale peu différencié compatible avec un carcinome à petites cellules du col utérin

**Figure 2:**
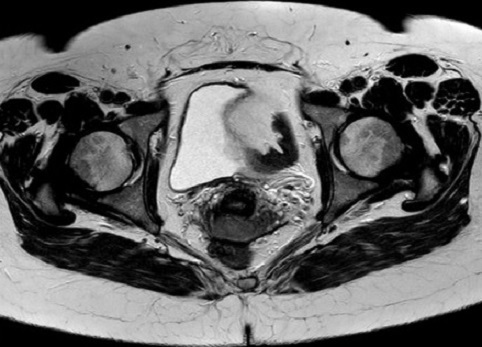
Coupe axiale de l’IRM pelvienne en séquence T2 montrant la masse tumorale de la berge externe du col utérin avec envahissement paramétrial gauche stade IIB selon la classification FIGO

L’étude immunohistochimique complémentaire effectuée sur coupes inclues en paraffine objective un intense marquage des cellules carcinomateuses à l’aide de l’anticorps anti-chromogranine A. Quelques cellules sont discrètement marquées par l’anticorps CD56. Ainsi l’aspect cyto-architecturale et immunohistochimique était compatible avec un carcinome à petites cellules du col utérin (Figure 1). L’imagerie par résonance magnétique pelvienne a retrouvé une volumineuse lésion tumorale de la berge externe du col utérin avec envahissement paramétrial gauche stade IIB selon la classification FIGO, associée à un envahissement ganglionnaire iliaque externe droit avec présence d’une volumineuse masse ganglio-tumorale (Figure 2).

Le PET scanner a retrouvé la masse intensément hypermétabolique du col utérin avec volumineuse adénopathie iliaque externe droite apparaissant isolée. L’indication retenue en réunion de concertation pluridisciplinaire était une radio-chimiothérapie concomitante à base de cisplatine 80mg/m^2^ à J1 associé à de l’étoposide 100mg/m^2^de J1 à J3, à raison d’une cure chaque 3 semaines, en concomitant avec de la radiothérapie à la dose de 48Gy en 30 séances sur les aires ganglionnaires lombo-aortiques, 60Gy en 30 séances sur les paramètres gauches, 60Gy en 30 séances sur les aires ganglionnaires iliaques externes et iliaques primitives, 48Gy en 30 séances sur le lit tumoral. La radiothérapie a été complétée par une curiethérapie à la dose de 24Gy en 5 fractions. L’évaluation post-thérapeutique a conclu à une stérilisation tumorale, avec un recul de 6 mois.

## Discussion

Le carcinome neuroendocrine est une tumeur maligne rare et agressive, se développant principalement aux dépens du poumon et du tractus digestif. Il ne représente que 3% des tumeurs du col utérin qui sont majoritairement des carcinomes épidermoïdes [1, 2]. Identifié pour la première fois en 1957 [3], son incidence réelle est probablement sous-estimée car décrit sous différentes terminologies telles que tumeur carcinoïde, carcinome à cellules argyrophile, apudome, carcinome à cellules en « grain d’avoine », carcinome neuroendocrine, carcinoïde atypique, carcinome à petites cellules indifférenciées ou carcinome à cellules intermédiaires [1-3]. En 1997 et dans un souci d’homogénéisation, Albores-Saavedra et al., ont proposé une classification des tumeurs neuroendocrines en quatre sous-types, à savoir les carcinomes neuroendocrines à petites cellules, les plus fréquents caractérisés par une activité mitotique élevée, une nécrose extensive, une invasion vasculaire et qui sont fréquemment associées aux papillomavirus humains. Les autres sous-types sont les carcinomes neuroendocrines à grandes cellules et les tumeurs neuroendocrines différenciées que sont les tumeurs carcinoïdes typiques et atypiques [4]. Durant les deux dernières décennies, et contrairement aux carcinomes épidermoïdes du col utérin, une augmentation de l’incidence des carcinomes neuroendocrines à petites cellules a pu être observée reflétant l’utilisation de cette terminologie commune, ce qui a par ailleurs permis, grâce à la publication de séries rétrospectives, de mettre en évidence plusieurs caractéristiques qui leurs sont propres.

Ces tumeurs surviennent à un âge médian de 42ans (20-87) [2-5], ce qui semble plus jeune que pour les carcinomes épidermoïdes du col utérin. La symptomatologie clinique est non spécifique le plus souvent la patiente consule pour des métrorragies provoquées des leucorrhées récidivante, et une masse pelvienne retrouvée à l’examen, rarement les patients présentent les caractéristiques cliniques et biochimiques d’une sécrétion hormonale (syndrome de cushing, syndrome carcinoïde, hypoglycémie, syndrome de sécrétion inappropriée d’hormone antidiurétique, hypocalcémie).

A la différence des carcinomes épidermoïdes du col utérin, les carcinomes neuroendocrines sont souvent diagnostiquées tardivement du fait de l’inefficacité des frottis cervico vaginaux dans le dépistage des carcinomes neuroendocrines à petites cellules [6, 7]. Le diagnostic repose sur l’étude histologique est surtout immunohistochimique par la mise en évidence d’au moins un marqueur neuroendocrine (synaptophysine, chromogranine A, énolase neurone spécifique) qui permet le diagnostic. Macroscopiquement la tumeur est volontiers endocervicale. Deux formes principales s’observent : la forme nodulaire massive peu rattachée à la muqueuse cylindrique et la tumeur basaloide multi micronodulaire.

A l’examen histologique les carcinomes neuroendocrines montrent un spectre histologique allant d’une tumeur carcinoïde typique ou atypique à un carcinome à petites cellules. Les cellules endocrines possèdent une diversité considérable de taille, d’argyrophilie, de colorations immunocytochimiques et de l’ultrastructure. Elles peuvent être identifiées en histochimie (coloration de Grimelius) en ultrastructure par la mise en évidence de granulations argyrophiles ou neurosecretoires, en immunocytochimie par une positivité à la NSE, à la chromogranine, à la synaptophysine et aux anticorps pour la gastrine, l’insuline ou par la production ectopique d’ACTH, de <#126>MSH, de sérotonine, d’histamine, d’amylose. Mannion et al ont comparé les caractéristiques microscopiques et le taux de survie des quatre catégories; les carcinomes à petites cellules étaient de plus mauvais pronostic présentant des similitudes avec les carcinomes à petites cellules du poumon. Ils sont caractérisés par un index mitotique élevé, une nécrose étendue, et une invasion lymphatique et vasculaire massive avec une forte association à l’HPV18 [8].

Vu la forte tendance à la dissémination régionale et à distance, le bilan doit comprendre une imagerie abdomino-pelvienne de préférence une imagerie par résonnance magnétique. Actuellement, et dans le but d’améliorer le staging ganglionnaire la TEP (tomographie par émission de positon a montré une supériorité dans cette indication tant au niveau pelvien que lombo-aortique; Permettant le double suivi des lésions cibles à la fois d’un point de vue morphologique et métabolique, elle devient l’outil de choix dès lors que l’on souhaite apprécier au mieux l’efficacité d’un traitement [9].

La stadification suit celle de toutes les tumeurs cervicales. Cependant, il est important de reconnaitre le risque accru d’invasion lymphatique et vasculaire et le taux élevé de récurrences extra pelviennes. Par exemple l’invasion lymphatique précoce des adénopathies locorégionales était objectivée dans 40% des stades IB des tumeurs à petites cellules de moins de 3 cm de diamètre. Dans 60% de ces tumeurs l’invasion vasculaire et lymphatique était constatée au moment du diagnostic. Le délai de récurrence est de 19.9 mois [10]. Les métastases sont plus communément osseuses, supra-claviculaires et pulmonaire. Le traitement des carcinomes neuroendocrines du col utérin est calqué sur celui des carcinomes épidermoïdes en prenant en considération les caractéristiques des tumeurs neuroendocrines du poumon.

Pour les tumeurs localisées stade I-IIA, le traitement locale ne suffit pas, deux auteurs ont rapporté des résultats décevants, Sheet et al, les premiers, ont retrouvé un taux de survie globale à trois ans de 16% et un taux de survie sans progression à cinq ans de 0 % [11]. Pour Sevin et al, ce dernier était de 36 % [12]. Des rechutes principalement hématogènes (67 à 90 % des cas) et ganglionnaires (34% des cas), une incidence élevée d’adénopathies au diagnostic (40-60 %), et une invasion vasculaire fréquente, sont autant de facteurs qui ont incité la majorité des auteurs à associer un traitement systémique au traitement local [13], Zivanovic et al. ont comparé rétrospectivement un traitement local seul (chirurgie) et un traitement local associé à une chimiothérapie adjuvante. Ils ont retrouvé un taux de survie sans récidive à trois ans de 83% pour les patientes ayant reçu une chimiothérapie à base de cisplatine et d’étoposide contre 0 % en cas de traitement local seul. Pour les tumeurs à un stade avancé, les métastases sont traitées par chimiothérapie combinée à base de platines. Alors que le taux de réponse initial est assez élevé (50-79%) la récurrence ou la chimiorésistance se développe. Alors une thérapie 2^ème^ ligne est mise en route par vincristine/doxorubicin/cyclophosphamide et topotecan.

Du fait du taux de dissémination métastatique précoce, certains auteurs ont préféré utiliser une chimiothérapie néoadjuvante, Chang a démontré une réponse complète de 6 sur 7 patientes ayant reçu le VAC/PE avant l’hystérectomie; cependant des résidus microscopiques étaient présents dans tous les cas. Une chimiothérapie adjuvante était nécessaire. Par conséquent 3 patientes étaient indemnes à 16, 45 et 56 mois de suivi. Lee et al n’ont par contre pas objectivé de bénéfice sur les 6 patientes ayant reçu une thérapie néo adjuvante [14].

En l’absence d’essais comparant radiothérapie et chirurgie, certains auteurs ont préféré les intégrer dans le cadre d’un traitement multimodal. En associant chirurgie, radiothérapie et chimiothérapie, Chan et al ont réussi à obtenir un taux de survie à cinq ans de 32%, ce qui est nettement supérieur à ceux rapportés dans les différentes séries. Les patientes survivantes à long terme étaient celles atteintes de tumeur de moins de 2 cm et ayant bénéficié d’une chirurgie radicale [15]. Pour les tumeurs localement évoluées (stades IIb–IV) et pour les patientes inopérables, une association de radiothérapie et de chimiothérapie est préconisée, selon le protocole d’Hoskins et al [16]. A ces stades, une chimiothérapie comportant au moins cinq cures de cisplatine et d’étoposide est associée à une meilleure probabilité de survie sans récidive et spécifique.

En cas de maladie métastatique ou de récidive, une chimiothérapie, comportant soit du cisplatine et de l’étoposide seuls, soit en alternance avec une chimiothérapie de type VAC (vincristine, adriamycine et cyclophosphamide) est indiquée. Les facteurs pronostic sont le stade clinique, la taille tumorale, la présence et le nombre d’adénopathies métastatiques, l’histologie à petites cellules et le tabac. Le stade clinique était le seul facteur prédictif de survie, 80% au stade I/II, et 38% au stade III/IV. Les sites de rechute à distance les plus communs sont l’os et le poumon (28%) plutôt que la rechute locale (13%) [17].

## Conclusion

Des essais cliniques multicentriques sont nécessaires pour essayer de déterminer un traitement univoque et efficace pour les carcinomes neuroendocrines à petites cellules du col utérin, afin d’améliorer la survie des patientes.

## Conflits d’intérêts

Les auteurs ne déclarent aucun conflit d’intérêt.
